# P-431. Epidemiologic profile of patients with HIV/AIDS in critical care, a Colombian cohort study

**DOI:** 10.1093/ofid/ofae631.631

**Published:** 2025-01-29

**Authors:** Juan José Castro Palacio, Laura Maria Serna Patiño, sara Penagos, Natalia Zapata, Juan pablo villa Franco, Juan Camilo Peláez, Carlos Andres Agudelo Restrepo, Alicia Hidron, sebastian Rivera, Silvana zapata, carlos Galeano

**Affiliations:** Universidad Pontificia Bolivariana, Medellin, Antioquia, Colombia; Universidad Pontificia Bolivariana, Medellin, Antioquia, Colombia; Hospital Pablo Tobon Uribe, Medellin, Antioquia, Colombia; Hospital Pablo Tobon Uribe, Medellin, Antioquia, Colombia; Hospital Pablo Tobon Uribe, Medellin, Antioquia, Colombia; Universidad Pontificia Bolivariana, Medellin, Antioquia, Colombia; Universidad Pontificia Bolivariana, Medellin, Antioquia, Colombia; Universidad Pontificia Bolivariana, Medellin, Antioquia, Colombia; Universidad Pontificia Bolivariana, Medellin, Antioquia, Colombia; Universidad de Antioquia, Medellin, Antioquia, Colombia; Universidad Pontificia Bolivariana, Medellin, Antioquia, Colombia

## Abstract

**Background:**

The underlying cause of mortality in patients with HIV/AIDS admitted to critical care units has been changing since the advent of highly active antiretroviral therapy (HAART). This study was done to describe the clinical and epidemiologic profile of patients with HIV in a low-middle income country in the HAART era

Baseline characteristics of 806 HIV admissions to ICU

**Figure:**
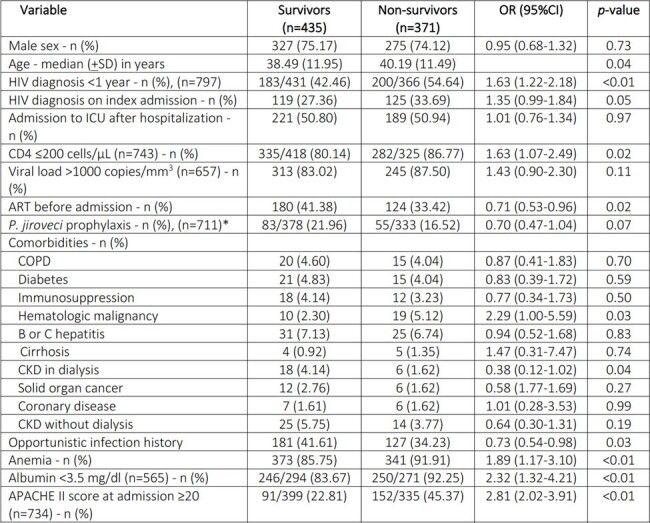

*Only patients with CD4 count ≤200 cel/ µL. Missing data in 21 of 743 patients with CD4 count data available and ≤200 cells/µL

ART: antiretroviral therapy. CKD: chronic kidney disease. COPD: chronic obstructive pulmonary disease.

**Methods:**

This was a retrospective cohort study which included adult patients with a diagnosis of HIV, admitted for at least 24 hours to the intensive care unit (ICU) in either of 5 hospitals in Medellín, Colombia, between January 2009 and December 2020. Demographic, clinical and laboratory data were collected. A descriptive analysis and an univariable analysis were done and missing data were imputed. A binomial logistic regression was performed with age and sex considered a priori as confounders; other variables were included in the multivariable analysis if *p*< 0.05.

Causes for ICU admission in HIV-positive patients

**Figure:**
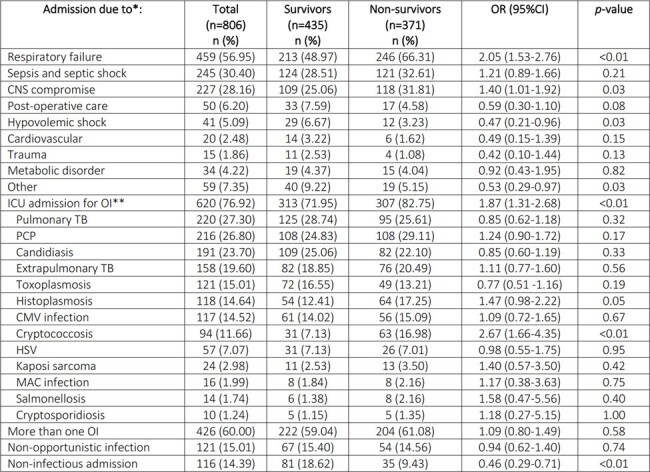

*Patients could have more than 1 cause of admission to ICU

**Patients could have more than 1 opportunistic infection at ICU admission

CMV: cytomegalovirus. CNS: central nervous system. HSV: herpes simplex virus. MAC: Mycobacterium avium complex. OI: opportunistic infection. PCP: Pneumocystis jiroveci. TB: tuberculosis.

**Results:**

During the study period, 806 admissions of HIV patients to the ICU were recorded. More than 80% of patients had a CD4 count of < 200 cells/µL. ICU admission for an opportunistic infection (OI) was more frequent in patients who died, compared to patients without an OI. OIs explained 76% of admissions (Table 1). The main cause for ICU admission was respiratory failure followed by sepsis and central nervous system (CNS) compromise (Table 2). A recent diagnosis of HIV (< 1 year), history of a hematologic malignancy, an APACHE II score ≥20, acute respiratory failure, CNS compromise as the cause of ICU admission and empiric use of amphotericin B were all associated to higher mortality among HIV patients admitted to the ICU (Table 3). A trend to a lower proportion of OIs as the cause for admission, correlated with a lower mortality trend throughout the years (close to 50% mortality in 2009 and to 15% in 2020 (Graph 1).

Variables associated with mortality in HIV infected patients admitted to the ICU

**Figure:**
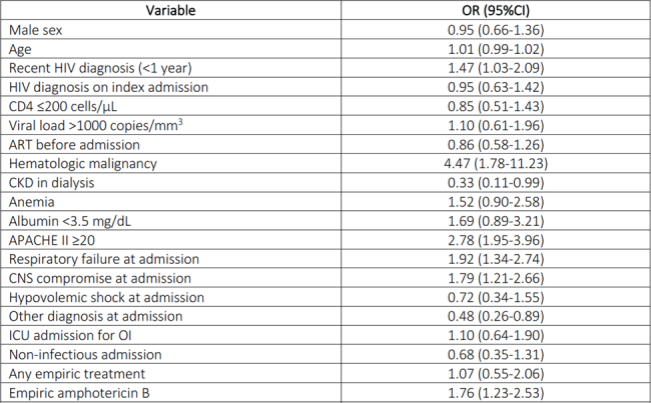

AKI: acute kidney injury. ART: antiretroviral therapy. CNS: central nervous system. OI: opportunistic infection. ORa: adjusted Odds ratio

**Conclusion:**

The main cause of mortality in VIH patients admitted to the ICU continues to be respiratory failure and sepsis secondary to OIs in low-middle income countries like Colombia. Mortality was associated to overall disease severity (respiratory failure, APACHE II score ≥20) and certain host conditions (hematologic malignancies and CNS compromise). Despite an epidemiologic profile typical for a low-middle income country, mortality has been decreasing consistently throughout the years.

Trends in mortality and type of admission diagnosis in HIV infected patients admitted to the ICU

**Figure:**
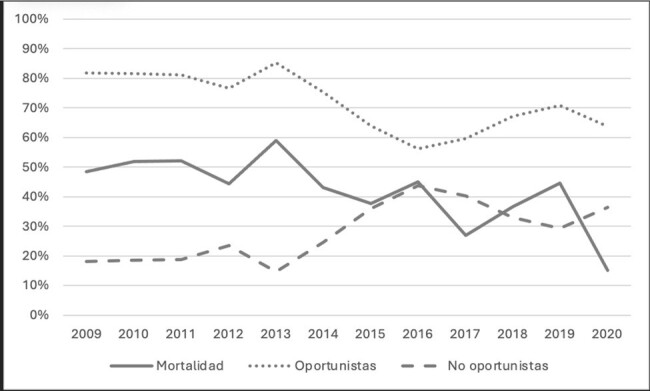

**Disclosures:**

**All Authors**: No reported disclosures

